# Comparison of Postpartum Health Care Use and Spending Among Individuals with Medicaid-Paid Births Enrolled in Continuous Medicaid vs Commercial Insurance

**DOI:** 10.1001/jamanetworkopen.2022.3058

**Published:** 2022-03-18

**Authors:** Sarah H. Gordon, Alex Hoagland, Lindsay K. Admon, Jamie R. Daw

**Affiliations:** 1 Department of Health Law, Policy, and Management, Boston University School of Public Health, Boston, Massachusetts; 2 Department of Economics, Boston University, Boston, Massachusetts; 3 Department of Obstetrics and Gynecology, University of Michigan Medical School, Ann Arbor; 4 Department of Health Policy and Management, Columbia University School of Public Health, New York, New York

## Abstract

**Importance:**

Postpartum Medicaid eligibility extensions are likely to shift enrollees from commercial to Medicaid coverage in the postpartum year; however, the potential implications for health care use and spending are unknown.

**Objective:**

To compare health care use and spending among individuals with a Medicaid-paid birth who had continuous Medicaid vs continuous commercial insurance during months 3 to 12 post partum.

**Design, Setting, and Participants:**

Cross-sectional study using linked all-payer claims, birth records, and income data for Medicaid-paid births in the Colorado All Payer Claims Database from 2014 to 2019 to estimate the association between continuous Medicaid vs commercial insurance and health care use and spending during months 3 to 12 post partum.

**Exposure:**

Continuous enrollment in Medicaid vs commercial insurance during months 3 to 12 post partum.

**Main Outcomes and Measures:**

Primary outcomes were the rate and number of primary care and outpatient visits, and total out-of-pocket spending during months 3 to 12 post partum. Secondary outcomes were the rate and number of emergency department visits and hospitalizations during months 3 to 12 post partum.

**Results:**

The 44 471 individuals in the sample had a mean (SD) age of 26.8 (5.50) years. Self-reported race and ethnicity included 1279 (2.9%) Asian individuals, 4028 (9.1%) Black individuals, 33 534 (75.4%) White individuals, as well as 5630 (12.7%) individuals of other race and ethnicity (American Indian or Alaskan Native; Other Pacific Islander; and unspecified). Of these, 19 337 (43.5%) self-identified as Hispanic individuals. The sample included 42 989 individuals continuously enrolled in Medicaid and 1482 individuals continuously enrolled in commercial insurance during months 3 to 12 post partum. Compared with those continuously enrolled in Medicaid, commercially insured enrollees were older (32.2% of commercial enrollees were between the ages of 30-39 vs 27.5% of Medicaid enrollees, *P* < .001), less likely to be Hispanic (38.9% in commercial vs 43.7% in Medicaid, *P* < .001) or born in the US (15.6% in commercial vs 19.6% in Medicaid, *P* < .001), and more likely to be married (62.8% in commercial vs 54.8% in Medicaid, *P* < .001), have completed college (32.9% in commercial vs 16.5% in Medicaid, *P* < .001), and initiated early prenatal care (79.7% in commercial vs 72.5% in Medicaid, *P* < .001). In multivariable models, individuals with commercial insurance were 2.46 percentage points (95% CI, 2.12-2.79 percentage points; *P* < .001) more likely to attend a primary care visit and had 0.81 (95% CI, 0.70-0.92; *P* < .001) additional primary care visits total during months 3 to 12 post partum. Individuals enrolled in commercial insurance were 7.92 percentage points (95% CI, −8.44 to −7.40 percentage points; *P* = .006) less likely to visit an emergency department compared with those enrolled in Medicaid. Total adjusted per person spending was $1110 (95% CI, $509-$1710; *P* < .001) higher, and total out-of-pocket spending per person was $796 (95% CI, $754-$838; *P* < .001) higher for those enrolled in commercial insurance vs Medicaid.

**Conclusions and Relevance:**

In this study, primary care use was higher and emergency department use was lower among those continuously enrolled in commercial vs Medicaid insurance during months 3 to 12 post partum. Medicaid rather than commercial insurance was associated with decreased exposure to out-of-pocket costs during months 3 to 12 postpartum for individuals with low income.

## Key Points

**Question:**

How do health care use and spending compare in the postpartum year for individuals with Medicaid vs commercial insurance?

**Findings:**

In this cross-sectional study of 44 471 Medicaid-paid births in Colorado, individuals enrolled in commercial insurance after their pregnancy-related Medicaid eligibility ended had higher rates of primary care and lower rates of emergency department use during months 3 to 12 post partum compared with those who remained enrolled in Medicaid. Total and out-of-pocket costs were significantly higher among those with commercial insurance compared with Medicaid.

**Meaning:**

Continuous Medicaid coverage was associated with lower rates of primary care use and less exposure to out-of-pocket costs.

## Teaser

This cross-sectional study of Medicaid-paid births in Colorado compares health care use and spending among individuals with continuous Medicaid coverage vs those with commercial insurance in the postpartum year.

## Introduction

The Medicaid program is a critical lever to improve postpartum health in the US because the program pays for 42% of all US births.^1^ Postpartum Medicaid extensions have been proposed as a policy to address postpartum maternal morbidity in the US because approximately one-third of postpartum mortality occurs between 7 days and 1 year post partum, with an increasing number of cases occurring late in the postpartum year.^2,3^ In March 2021, Congress passed the American Rescue Plan (ARP) Act, which included a state option to use federal matching funds to extend pregnancy-related Medicaid eligibility from 60 days post partum to up to 1 year after birth. In November 2021, the US House of Representatives passed the Build Back Better Act, which would make a 1-year postpartum Medicaid eligibility extension mandatory for all states. As of January 2022, Virginia, New Jersey, Missouri, Illinois, and Georgia have approved 1115 waivers to extend postpartum coverage for at least a portion of Medicaid enrollees, and at least 20 other states have taken action to implement an extension.^4^

In all states, pregnancy-related Medicaid eligibility limits exceed income-based eligibility limits for other Medicaid eligibility categories, such as low-income parents or adults.^5,6^ The 60-day duration of postpartum pregnancy-related Medicaid eligibility creates an eligibility “cliff” in which individuals who are ineligible to remain enrolled in Medicaid through one of these alternative eligibility pathways must enroll in commercial or other insurance or become uninsured. Individuals who lose Medicaid eligibility can enroll in marketplace coverage during a special enrollment period 60 days after the birth of their child or obtain commercial insurance.

Approximately 20% of individuals with Medicaid-paid births subsequently enroll in commercial coverage, indicating that postpartum Medicaid extensions could result in a shift from commercial coverage to Medicaid during months 3 to 12 post partum.^7^ Evidence is needed to understand the implications of potentially shifting individuals from commercial to Medicaid coverage during the postpartum year under proposed extensions of postpartum Medicaid coverage. The objective of this study was to compare health care use and spending among individuals with Medicaid-paid births who were continuously enrolled in Medicaid vs commercial insurance during months 3 to 12 post partum. Using linked all-payer claims, birth records, and income data from Colorado, we assessed the association between Medicaid vs commercial insurance and the use of primary and outpatient care, emergency departments (EDs), inpatient hospitalization, and total and out-of-pocket spending during the postpartum year.

## Methods

### Data and Sample

We used a linked data set composed of the Colorado All Payer Claims Database (APCD), statewide birth records, and income data. The APCD includes health care claims from the state’s largest commercial health insurers, Health First Colorado claims (Colorado’s Medicaid program), Connect for Health Colorado (Colorado’s individual health insurance marketplace), non–Employee Retirement Income Security Act commercial self-insured plans, and commercial Employee Retirement Income Security Act self-insured plans that submit claims on a voluntary basis.^8^ Birth records were linked with the APCD by the Colorado Department of Public Health and Environment, and individual-level income data for Medicaid enrollees were linked to the APCD by the Colorado Department of Health Care Policy and Financing (eAppendix 1 in the Supplement). This study was approved by the Boston University School of Public Health’s institutional review board. Informed consent was waived because we used a deidentified secondary data source. This study followed the Strengthening the Reporting of Observational Studies in Epidemiology (STROBE) reporting guidelines.^9^ Data on race and ethnicity categories were ascertained from the birth record, which assesses race and ethnicity via maternal self-report.

In Colorado, pregnant individuals are eligible for Medicaid or the Children’s Health Insurance Program if their incomes fall below 265% of the federal poverty level (FPL). To qualify for Medicaid or the Children’s Health Insurance Program as a parent, caretaker, or an adult with low income, household income must fall at or below 138% FPL. Our sample consisted of individuals older than 19 years with Medicaid-paid births between 2014 and 2018 who were continuously enrolled in Medicaid or commercial insurance during months 3 to 12 of the year following the date of delivery. We excluded 2019 births so that we observed 1 year of postpartum follow-up for all births. We excluded individuals who did not have a corresponding birth record or were missing income information, those with records in which the date of delivery was not within the period that the state Medicaid agency indicated the mother was enrolled in Medicaid, and those with records in which the mother and infant shared the same Medicaid identification (eAppendix 2 in the Supplement). We restricted the sample to individuals who were continuously enrolled in insurance during months 3 to 12 post partum (60.5% of the sample) to capture all health care use and costs that occurred during this period.

### Measures

The primary exposure variable was continuous enrollment in Medicaid vs commercial insurance during months 3 to 12 post partum, measured as mutually exclusive continuous enrollment in either type of coverage for the full 9 months. Medicaid coverage does not end until the last day of the month 60 days after childbirth, thus months 3 to 12 are the period to which postpartum Medicaid extensions under the ARP would apply.

We assessed the rate and number of health care use that occurred during this same period (months 3-12 post partum). We defined postpartum outpatient visits as all visits with place of service or revenue codes indicating an outpatient setting claim code, excluding laboratory claims and claims that occurred as part of an ED visit or inpatient hospitalization but including prenatal visits for a subsequent pregnancy. We defined primary care visits as the subset of outpatient visits that included any *Current Procedural Terminology* codes for a preventive office visit as well as those with *International Statistical Classification of Diseases and Related Health Problems, Tenth Revision* diagnosis codes for a general adult medical examination, such as well-woman examinations and postpartum care. We examined the number of outpatient and primary care visits as well as the probability of having at least 1 visit during months 3 to 12 post partum. Secondary outcomes included the number and rate of ED visits and hospitalizations. We defined ED visits as any claim with an ED place of service or revenue code. In addition, hospitalizations were defined using place of service or revenue codes indicating an inpatient setting and limited to lengths of stay greater than 24 hours (eAppendix 3 in the Supplement has the full list of codes).

Our primary spending outcome was total out-of-pocket spending per person in months 3 to 12 after childbirth for individuals with Medicaid vs commercial insurance, defined as the sum of enrollee coinsurance, copayment, and deductible payments. We also measured total out-of-pocket spending per person by service type (eg, outpatient visit, primary care visit, ED visit, and hospitalization) and total health care spending per person for all care delivered 3 to 12 months post partum and by service type calculated as the sum of insurer-paid amounts and enrollee out-of-pocket spending. These outcomes did not include spending on outpatient prescription drugs. To accommodate the highly skewed distribution of spending, we truncated data for individuals with total medical spending in the 99th percentile or greater from our primary sample; we report results using the untrimmed sample in eAppendix 4 in the Supplement. All spending measures were computed in 2020 US dollars using the medical component of the Consumer Price Index.

We selected covariates based on previous studies that indicated certain characteristics, including maternal age, race, ethnicity, education, and birth origin, were associated with insurance type.^10,11,12,13,14^ We used the linked birth record to measure age, race, ethnicity, education, marital status, maternal nativity (US vs non-US born), prenatal care use (first trimester prenatal care and number of visits), and chronic disease status prior to pregnancy (individuals diagnosed as having hypertension, diabetes, or obesity before pregnancy). We also measured rates of preterm delivery, cesarean delivery, and a composite measure of antenatal and delivery-related complications, including gestational diabetes, gestational hypertension, eclampsia, hemolysis, elevated liver enzymes, low platelet count syndrome, multiple gestation, infant intensive care unit admission, blood transfusion, uterine rupture, unplanned cesarean hysterectomy, and third- or fourth-degree lacerations. Covariate data were missing for less than 4% of enrollees (eAppendix 5 in the Supplement). We used Medicaid enrollment data provided by Colorado’s state Medicaid agency to measure postpartum income. Because incomes may fluctuate during pregnancy and the postpartum period, we defined a target income assignment date as the month after 60 days post partum. The selection of this point provided an updated income for the largest number of enrollees (58% of the sample). For the remainder of the sample, we looked backward from the target assignment date (month after 60 days post partum) and assigned the closest recorded income.

### Statistical Analysis

We conducted descriptive analyses comparing baseline covariates among individuals with Medicaid-paid births who were continuously enrolled in Medicaid vs commercial insurance during months 3 to 12 post partum using χ^2^ tests for binary and categorical variables and 2-sided *t* tests for continuous variables. We calculated unadjusted differences in mean health care use and spending over months 3 to 12 post partum among births to individuals enrolled in Medicaid vs commercial insurance and plotted mean, unadjusted monthly out-of-pocket spending per person over months 3 to 12 post partum. We estimated adjusted differences using multivariable regression models controlling for age, race, ethnicity, nativity, marital status, education, prepregnancy chronic conditions, number of prenatal visits, trimester of prenatal care initiation, delivery complications, cesarean sections, income, and year of birth. Models were estimated using Newton-Raphson methods (via the glm command in Stata [Stata Corp]) with a negative binomial distribution and a log link for health care use outcomes, a logit link for health care use likelihood, and a gaussian distribution for spending outcomes. As a sensitivity analysis, spending outcomes were also estimated using a negative binomial distribution.

Data analyses were performed using Stata MP, version 16.1. Differences were considered to be statistically significant if *P* < .05. Statistical significance was assessed using 2-sided tests.

## Results

The sample included 44 471 individuals with births financed by Colorado’s Medicaid program from 2014 to 2018 who were continuously enrolled in insurance in the postpartum year (only 6.7% of the sample was enrolled in Medicaid managed care; these individuals were not excluded). All the individuals in the sample were women with a mean (SD) age of 26.8 (5.50) years. Self-reported race and ethnicity included 1279 (2.9%) Asian individuals, 4028 (9.1%) Black individuals, 33 534 (75.4%) White individuals, as well as 5630 (12.7%) individuals of other race and ethnicity (American Indian or Alaskan Native; Other Pacific Islander; and unspecified); of these, 19 337 (43.5%) self-identified as Hispanic individuals. A total of 42 989 were continuously enrolled in Medicaid and 1482 were continuously enrolled in commercial insurance 3 to 12 months post partum. On average, the commercially insured sample was older and less likely to be Hispanic or born in the US; 32.2% of commercial enrollees were between the ages of 30 and 39 years vs 27.5% of Medicaid enrollees (*P* < .001). A total of 38.9% of the commercially insured sample was Hispanic compared with 43.7% of the Medicaid-insured sample (*P* < .001). A total of 15.6% of the commercially insured sample was born outside the US compared with 19.6% of the Medicaid-insured sample (*P* < .001). In the commercially insured sample, 62.8% of individuals were married vs 54.8% in the Medicaid-insured population (*P* < .001), and 32.9% of the commercially insured sample had completed college compared with 16.5% of the Medicaid-enrolled sample (*P* < .001) (Table 1). Individuals enrolled in commercial insurance were also more likely to have initiated prenatal care in the first trimester (80% in commercial insurance vs 72.5% in Medicaid; *P* < .001) and to have higher incomes. Differences in race (except for Asian race), antenatal and delivery-related complications, and cesarean birth between the 2 insured samples were not statistically significant.

**Table 1.** Characteristics of Individuals with Medicaid-Financed Births Continuously Enrolled in Medicaid vs Commercial Insurance During Months 3 to 12 Post Partum^a^

Characteristic	3 to 12 mo Postpartum individuals, No. (%)		*P* value
	Medicaid (n = 42 989)	Commercial (n = 1482)	
Maternal age, y			
18-29	30 325 (70.5)	966 (65.2)	<.001
30-39	11 808 (27.5)	477 (32.2)	<.001
≥40	852 (2.0)	39 (2.6)	.12
Maternal race			
Asian	1215 (2.8)	64 (4.3)	.005
Black	3911 (9.1)	117 (7.9)	.09
White	32 417 (75.6)	1117 (75.4)	.92
Other^b^	5446 (12.7)	184 (12.4)	.85
Maternal Hispanic ethnicity	18 760 (43.7)	577 (38.9)	<.001
Born outside the US	8408 (19.6)	231 (15.6)	<.001
Married	23 543 (54.8)	931 (62.8)	<.001
Education			
High school	34 135 (79.5)	1387 (93.6)	<.001
College	7081 (16.5)	488 (32.9)	<.001
Prepregnancy chronic conditions	12 026 (28.0)	409 (27.6)	.73
No. of prenatal visits	10.43	10.67	.02
First trimester prenatal care initiation	31 124 (72.5)	1181 (79.7)	<.001
Delivery complications	5078 (11.8)	199 (13.4)	.08
Cesarean delivery	10 348 (24.1)	342 (23.1)	.36
Income at 60 d post partum, % FPL			
0-100	33 363 (77.6)	733 (49.5)	<.001
101-138	4790 (11.1)	196 (13.2)	.02
139-200	2939 (6.8)	315 (21.3)	<.001
201-265	1004 (2.3)	143 (9.7)	<.001
266-300	180 (0.4)	30 (2.0)	<.001
301-400	169 (0.4)	25 (1.7)	<.001

Abbreviation: FPL, federal poverty level.

^a^ Frequency and relative proportions of all binary covariates are reported for both groups; only frequencies are reported for continuous covariates. All *t* tests are performed on proportions when covariate is binary.

^b^ Other includes American Indian or Alaskan Native, Other Pacific Islander, and unspecified race.

Adjusting for these factors, individuals continuously enrolled in commercial insurance were 2.46 percentage points (95% CI, 2.12-2.79 percentage points; *P* < .001) more likely to have a primary care visit, 7.12 percentage points (95% CI, 6.39-7.84 percentage points; *P* < .001) more likely to have an outpatient visit, and 7.92 percentage points (95% CI, −8.44 to −7.40 percentage points; *P* = .006) less likely to have an ED visit compared with those continuously enrolled in Medicaid during months 3 to 12 post partum (Table 2). Those enrolled in commercial insurance were also 0.48% less likely to have a hospitalization during this period compared with individuals enrolled in Medicaid, although results for hospitalizations should be interpreted with caution because the total number of hospitalizations was low among the commercially insured sample (n = 13). On average, those continuously enrolled in commercial insurance had 0.16 (95% CI, 0.10-0.22; *P* < .001) more outpatient visits, 0.81 (95% CI, 0.70-0.92; *P* < .001) additional primary care visits, and 0.14 (95% CI, −0.24 to −0.04; *P* = .008) fewer ED visits.

**Table 2.** Use of Outpatient and Primary Care Services, Emergency Departments, and Hospitalizations During Months 3 to 12 Post Partum Among Individuals with Medicaid-Financed Births Continuously Enrolled in Medicaid vs Commercial Insurance^a^

Variable	Medicaid enrollment	Commercial enrollment	Unadjusted difference (95% CI)	Adjusted difference (95% CI)^**b**^
No. of births	42 989	1482	NA	NA
**Patients with any health care use, No. (%)**				
Outpatient visits	31 683 (73.7)	1175 (79.3)	5.65 (3.54 to 7.76)	7.12 (6.39 to 7.84)
Primary care visits	4944 (11.5)	218 (14.7)	3.20 (1.38 to 5.03)	2.46 (2.12 to 2.79)
ED visits	12 080 (28.1)	298 (20.1)	−8.00 (−10.09 to −5.92)	−7.92 (−8.44 to −7.40)
Hospitalizations	731 (1.7)	12 (0.8)	−0.91 (−1.38 to −0.43)	−0.48 (−0.51 to −0.45)
**Mean health care use, No.**				
Outpatient visits	6.65	6.50	−0.15 (−0.59 to 0.29)	0.16 (0.10 to 0.22)
Primary care visits	0.18	0.32	0.14 (0.07 to 0.21)	0.81 (0.70 to 0.92)
ED visits	0.59	0.40	−0.19 (−0.25 to −0.13)	−0.14 (−0.24 to −0.04)
Hospitalizations	0.02	0.01	−0.01 (−0.01 to −0.00)	−0.40 (−0.91 to 0.10)
**Mean total spending per person, $**				
Total costs	4207	4715	508 (−133 to 1149)	1110 (509 to 1710)
Outpatient visits	1952	2206	254 (−127 to 634)	360 (21 to 699)
Primary care visits	40	61	21 (3 to 45)	29 (7 to 51)
ED visits	1433	2137	704 (255 to 1154)	1086 (805 to 1367)
Hospitalizations	710	300	−410 (−606 to −215)	−309 (−638 to 19)
**Mean out-of-pocket spending per person, $**				
Total out-of-pocket	18	801	783 (620 to 946)	796 (754 to 838)
Outpatient visits	8	370	361 (270 to 452)	362 (341 to 383)
Primary care visits	0.12	5	5 (3 to 8)	5 (5 to 6)
ED visits	6	381	375 (259 to 492)	387 (359 to 415)
Hospitalizations	4	36	32 (−30 to 95)	34 (15 to 54)

Abbreviation: ED, emergency department.

^a^ Spending is measured in 2020 US dollars, inflated using the medical care services component of the consumer price index. Health care use variables are measured as a binary outcome for any visit of that type during the 3 to 12 months post partum and in counts (at the visit level). Spending measures (total and out of pocket) are reported as unconditional per person averages during the 3- to 12-month period. Sample is trimmed with the top 1% of individuals with the highest levels of billed spending removed. Billed spending measures the sum of insurer and consumer payments.

^b^ The adjusted differences column reports regression-adjusted coefficients and 95% confidence intervals for each variable. Each coefficient is the estimated coefficient on the treatment dummy (where 1 = continuously enrolled in commercial; 0 = continuously enrolled in Medicaid). Regression covariates include all covariates in Table 1 and year fixed effects. Models are estimated using Newton-Raphson methods (using the glm command in Stata [StataCorp]) with a logit link for use likelihood, a negative binomial distribution, and a log link for use outcomes and a gaussian distribution for spending outcomes.

The Figure displays unadjusted total out-of-pocket spending per person by month during the last 10 months of the postpartum year. Total adjusted out-of-pocket spending per person was $796 (95% CI, $754-$838; *P* < .001) higher in the commercial insurance sample compared with the Medicaid sample, $362 (95% CI, $341-$383; *P* < .001) higher for outpatient visits, $5 (95% CI, $5-$6; *P* < .001) higher for primary care visits, $387 higher for ED visits (95% CI, $359-$415; *P* < .001), and $34 (95% CI, $15-$54; *P* < .001) higher for hospitalizations.

**Figure.**
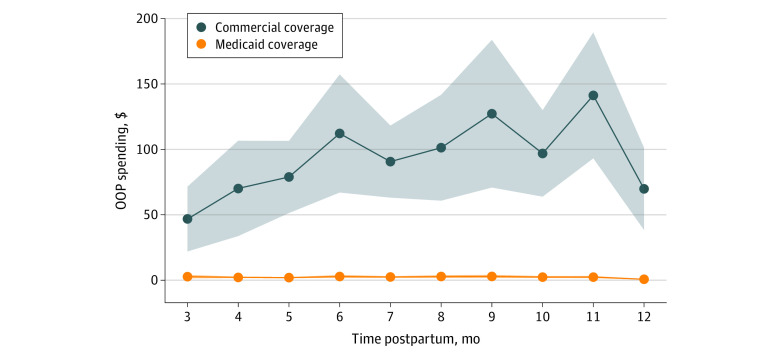
Mean Unadjusted Out-of-Pocket (OOP) Spending Per Person in Medicaid vs Commercial Insurance Groups During Months 3 to 12 Post Partum Figure shows mean total OOP spending per person for all health care encounters during months 3 to 12 post partum in commercial vs Medicaid insurance groups among individuals with Medicaid-paid births in Colorado from 2014 to 2018. The shading indicates the 95% CIs for each monthly data point.

Unadjusted total spending per person was $4207 in the Medicaid-enrolled sample compared with $4715 in the commercial insurance–enrolled sample (*P* = .06). After multivariable adjustment, individuals with commercial insurance had $1110 (95% CI, $509-$1710; *P* < .001) higher total spending per person compared with those with Medicaid. Adjusted spending differences per person were $360 (95% CI, $21-$699; *P* = .04) higher for outpatient visits, $29 (95% CI, $7-$51; *P* = .01) higher for primary care visits, and $1086 (95% CI, $805-$1367; *P* < .001) higher for ED visits among those continuously enrolled in commercial vs Medicaid insurance. No significant spending differences were observed for hospitalizations. Spending results on the untrimmed sample and applying a negative binomial distribution were similar, although spending differences were generally larger in magnitude (eAppendix 4 in the Supplement).

## Discussion

In this comparison of health care use and costs between individuals with Medicaid-paid births who were continuously enrolled in Medicaid vs commercial insurance during months 3 to 12 post partum, we found that those enrolled in commercial insurance had higher use of outpatient and primary care and lower ED use compared with those enrolled in Medicaid. We also found that total health care costs were significantly higher in the group with commercial insurance compared with Medicaid and that enrollees in commercial insurance paid much higher out-of-pocket costs across multiple service types.

These findings have important implications for state policy makers deciding whether to extend postpartum pregnancy-related Medicaid eligibility beyond 60 days under ARP and federal policy makers deciding whether to make the postpartum extension a mandatory coverage category, as proposed in the Build Back Better Act. On one hand, findings of the present study suggest that individuals who shift from commercial insurance to Medicaid as a result of postpartum Medicaid extensions will face significantly less exposure to out-of-pocket costs than those enrolled in commercial insurance owing to cost-sharing limits in Medicaid. Lower out-of-pocket costs may promote better access to care because previous studies have found that even small amounts of cost sharing can deter low-income patients from seeking necessary health care services.^15,16^ Medicaid may also offer more comprehensive benefits compared with some commercial plans.^17^ On the other hand, commercial insurance appears to be associated with greater use of outpatient care compared with Medicaid. This finding could be owing to greater availability of clinicians who accept commercial insurance or the fact that those who switch to commercial insurance may be motivated to seek a new physician. Alternatively, although we adjusted for many relevant covariates, those who successfully enroll in commercial insurance may be more motivated to engage with the health care system and therefore more likely to use outpatient care than those who remain enrolled in Medicaid.

The observed differences in health care use are consistent with previous research that has found higher use of outpatient preventive services and lower ED use among individuals enrolled in commercial plans compared with those enrolled in Medicaid.^18,19,20^ These findings could reflect higher barriers to ED care in commercial insurance or better access to preventive care in commercial plans. Differences in use of primary care have ambiguous implications for clinical practice because there are no clear guidelines for the recommended number of primary care visits in the postpartum year. Future research is needed to explore whether these differences in use are meaningfully associated with health outcomes. Rates of ED use were high in both the Medicaid and commercial insurance groups, suggesting that this is a common source of care for the postpartum population.

Differences in total spending likely reflect lower prices for medical services paid by Medicaid compared with commercial insurance plans.^21^ Under the ARP option and proposed in the Build Back Better Act, states receive the full federal Medicaid matching rate for the postpartum extension population, ranging from 56.2% to 84.5%, depending on the state.^22^ Overall, we found that per capita Medicaid spending for the postpartum sample during months 3 to 12 post partum was approximately $4200, less than per capita annual spending for the Medicaid expansion sample, for which the median was $6673 across 17 states in 2018.^23^ Although the extension of postpartum Medicaid eligibility imposes a cost to state budgets, financial implications will differ by whether the state already expanded Medicaid to all adults with incomes less than 138% FPL; the proportion of individuals with a Medicaid-paid birth that will be newly eligible for postpartum Medicaid is considerably higher in states that have not expanded.^24^ Regarding federal spending, the Congressional Budget Office estimates that if all states adopted a 12-month postpartum coverage provision, it would cost the federal government $1.2 billion over 10 years.^25^

### Strengths and Limitations

Two strengths of our analysis are that the study sample is limited to those with Medicaid-paid births with incomes less than 265% FPL, a more homogenous group than comparing Medicaid-paid births with commercial-paid births, and a primary factor driving commercial enrollment is Medicaid eligibility policy (the loss of Medicaid coverage 60 days post partum for those with incomes greater than 138% FPL) rather than characteristics of individuals. However, we cannot assume that if the commercially insured group were shifted into Medicaid for months 3 to 12 post partum, they would have the same use patterns as those continuously enrolled in Medicaid.

This study has several limitations. First, we relied on data from Colorado, which may limit generalizability, particularly to states that have not expanded Medicaid or rely heavily on Medicaid managed care. However, Colorado has Medicaid eligibility income limits similar to other Medicaid expansion states that are considering postpartum extensions.^5,6,26^ Furthermore, the linked database of all-payer claims, income data, and birth records was only available from Colorado. Second, we recognize the potential for unmeasured confounding between those who maintain continuous enrollment in Medicaid vs enroll in 10 months of continuous commercial insurance during the postpartum year.^27^ Although we control for prepregnancy health status and delivery complications, we cannot rule out selection bias caused by individuals who are more motivated to obtain health insurance, perhaps owing to a health event or preexisting illness.

Third, owing to the sample size for individuals continuously enrolled in commercial insurance, we were unable to conduct subgroup analyses by type of commercial coverage (eg, marketplace vs commercial plans) or by race and ethnicity. Fourth, because we used claims data, we were unable to assess outcomes among those who became uninsured post partum, an important area for future work. In addition, future work should investigate the association between postpartum insurance type and receipt of specific services, such as contraception and mental health care, as well as longer-term outcomes, such as short interpregnancy interval birth rates.

## Conclusions

The ARP provided states with an option to extend pregnancy-related Medicaid eligibility up to 1 year following childbirth. Our findings suggest that continuous Medicaid coverage is associated with lower rates of primary care and higher ED use and that providing continuous postpartum Medicaid eligibility could reduce total and out-of-pocket spending in the postpartum year.

## Notes

Supplement. **eAppendix 1.** Description of Data Linkage **eAppendix 2.** Sample Flow Chart **eAppendix 3.** Outcome Definitions with Codes **eAppendix 4.** Health Care Use and Spending Among Individuals in Commercial Versus Medicaid Insurance 3-12 Months Postpartum, Estimates Using Untrimmed Spending Sample **eAppendix 5.** Rates of Missing Covariate Data; Linked Colorado All Payer Claims Database, Birth Records, and Income, 2016-2019
