# Supplementary material for: Comparison of Postpartum Health Care Use and Spending Among Individuals with Medicaid-Paid Births Enrolled in Continuous Medicaid vs Commercial Insurance
Source: JAMA Netw Open. 2022 Mar 18;5(3):e223058. doi: 10.1001/jamanetworkopen.2022.3058 (PMC8933732; doi:10.1001/jamanetworkopen.2022.3058)
Supplement: Supplement. — eAppendix 1. Description of Data Linkage eAppendix 2. Sample Flow Chart eAppendix 3. Outcome Definitions with Codes eAppendix 4. Health Care Use and Spending Among Individuals in Commercial Versus Medicaid Insurance 3-12 Months Postpartum, Estimates Using Untrimmed Spending Sample eAppendix 5. Rates of Missing Covariate Data; Linked Colorado All Payer Claims Database, Birth Records, and Income, 2016-2019 [file jamanetwopen-e223058-s001.pdf]

Supplementary Online Content

Gordon SH, Hoagland A, Admon LK, Daw JR. Comparison of postpartum health care use and spending among individuals with Medicaid-paid births enrolled in continuous Medicaid vs commerical insurance. *JAMA Netw Open*. 2022;5(3):e223058. doi:10.1001/jamanetworkopen.2022.3058

eAppendix 1. Description of Data Linkage

eAppendix 2. Sample Flow Chart

eAppendix 3. Outcome Definitions with Codes

eAppendix 4. Health Care Use and Spending Among Individuals in Commercial Versus Medicaid Insurance 3-12 Months Postpartum, Estimates Using Untrimmed Spending Sample

eAppendix 5. Rates of Missing Covariate Data; Linked Colorado All Payer Claims Database, Birth Records, and Income, 2016-2019

This supplementary material has been provided by the authors to give readers additional information about their work.

© 2022 Gordon SH et al. *JAMA Network Open*.

eAppendix 1. Description of Data Linkage

Data Sources

Data for this study were obtained from three sources: the Colorado All Payer Claims Database which includes a supplemental file with information about Marketplace plans, individual-level income information for Health First Colorado enrollees (Colorado's Medicaid program), and birth records for all Colorado births. The APCD was obtained from the Center for Improving Value in Health Care (CIVHC), a non-profit organization which manages the APCD on behalf of the state. Individual-level income data were obtained from the Department of Health Care Policy and Financing, the state agency which oversees the Medicaid program. Birth records were obtained from the Colorado Department of Public Health and Environment.

The Colorado APCD receives health care claims from the state's largest commercial health insurers, covering 34% of commercially-insured lives in Colorado, comprised of non-ERISA commercial self-insured plans, and commercial ERISA self-insured plans that submit claims on a voluntary basis. Currently it is estimated that the APCD includes 25% of self-insured commercial lives in the state. This exclusion is due to the 2016 Gobeille versus Liberty Mutual Supreme Court decision that ruled that self-insured ERISA plans cannot be mandated to submit their claims to all payer claims databases. The APCD contains 100% complete Health First Colorado (Colorado's Medicaid Program) and CHIP claims. However, we may be unable to observe some transitions to commercial insurance after childbirth due to the exclusion of ERISA self-insured plans in the APCD.[1] However, the majority of enrollees in these plans work at larger firms and are typically high-earners, which may limit the extent to which women below 265% FPL transition to these unobserved plans.

Data Linkages

The linkage of the Colorado APCD with individual-level Medicaid enrollee income as a percentage of the federal poverty level was conducted in-house by the Colorado Department of Health Care Policy and Financing (HCPF). Income and eligibility variables are standard fields in Medicaid claims data prior to submission to the APCD. HCPF personnel added these fields back into the claims data using identified Medicaid enrollee numbers, which were then deidentified and returned to the research team linked with the APCD. The match rate between the sample of women with Medicaid-financed births identified in the APCD and the income file was 98.9%.

The linkage of the Colorado APCD with the birth records was conducted by CDPHE vital records staff using a deterministic matching process based on identifiable fields unique to APCD and the birth record: social security number (SSN), dates of birth (DOB), first name, and last name. CDPHE has used this approach to link other vital records data to administrative billing data. 96.1% of births identified in the APCD were matched with a corresponding birth record.

© 2022 Gordon SH et al. *JAMA Network Open*.

eAppendix 2. Sample Flow Chart

We generated our initial sample from all Medicaid-financed delivery hospitalizations in the Colorado APCD, from 2014-2018 (2019 births were excluded to observe a 12-month postpartum follow-up period for all births). Some birth record fields in APCD have been demonstrated to have poor accuracy in identifying birth payers; hence, we use administrative billing data rather than the birth record to identify birth payer.

We limited attention to births to women over 18 years old, as those younger than this qualify for Medicaid as children, with distinct eligibility criteria from adult women. We also excluded records for which the date income was recorded fell outside the dates of Medicaid eligibility provided by the state Medicaid agency and records with missing income data.

We then merged this file with the birth record data, retaining all exact or near-exact matches. To prevent issues arising from potentially conflated maternal and child IDs, we dropped records for which neonatal and postpartum services were linked to the same ID. Finally, to ensure unbiased estimates of utilization and spending, we required continuous enrollment in either Medicaid or commercial insurance coverage for at least 9 months postpartum.

© 2022 Gordon SH et al. *JAMA Network Open*.

Colorado Births CONSORT Flow Diagram, 2014-2018

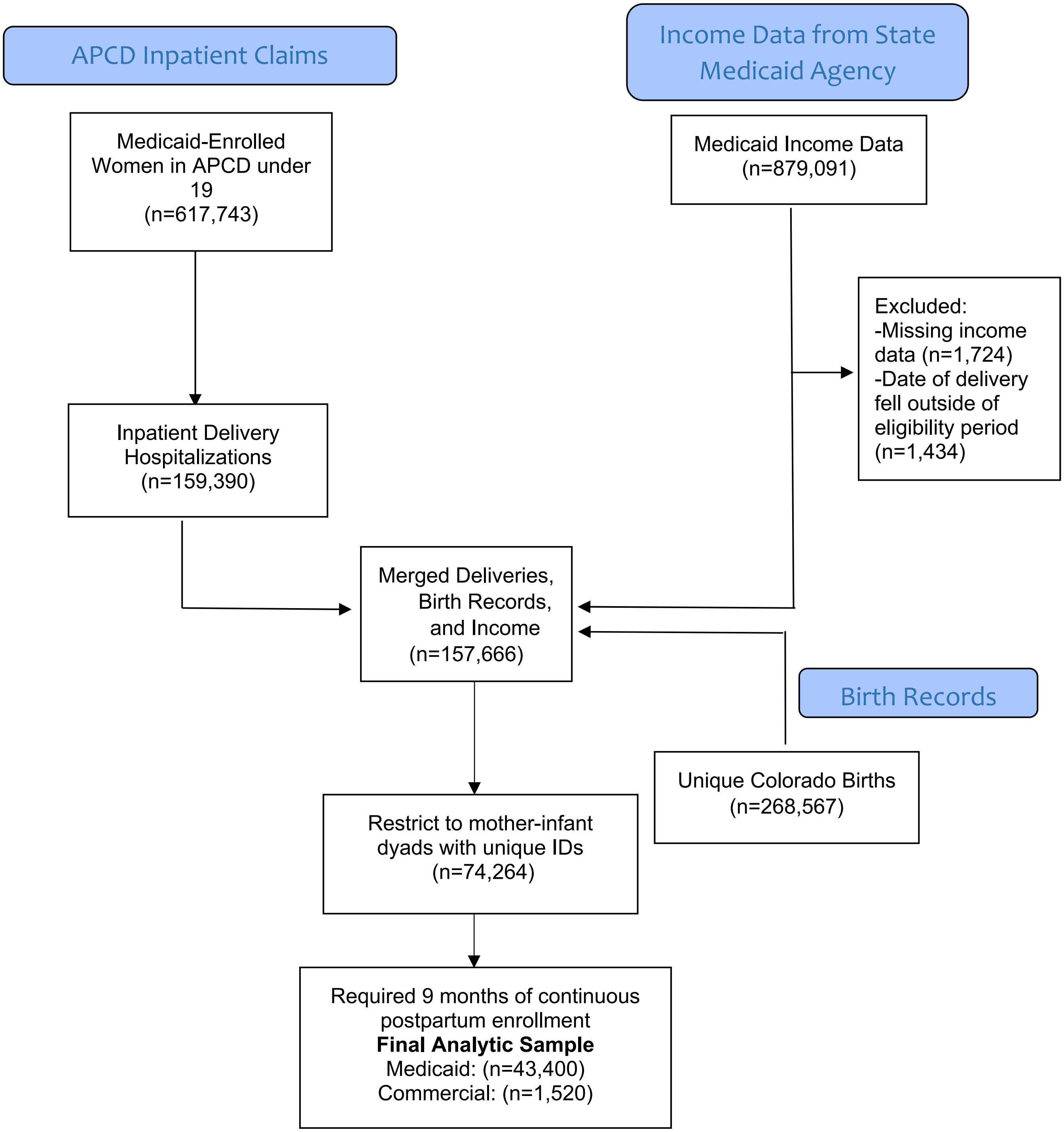

*Sample sizes refer to unique births in each data set.

© 2022 Gordon SH et al. *JAMA Network Open*.

eAppendix 3. Outcome Definitions with Codes

Newborn deliveries are identified using two data sources. Deliveries are isolated in the all-payer claims database using the ICD-10-CM diagnosis codes (or corresponding ICD-9-CM diagnosis codes) and CPT-4 delivery procedures listed in Table A2.1. We complement this approach with birth certificate data to ensure accurate measurement of the date of delivery.

ICD-10-CM	CPT-4
O80,	1960,
O80.0,	1961,
O80.1,	59400,
O80.8,	59409,
O80.9,	59410,
O82,	59412,
O82.0,	59414,
O82.1,	59510,
O82.2,	59514,
O82.8,	59515,
O82.9,	59610,
Z37,	59612,
Z37,	59614,
Z37.0,	59618,
Z37.1,	59620,
Z37.2,	59622
Z37.3,	
Z37.4,	
Z37.5,	
Z37.50,	
Z37.51,	
Z37.52,	
Z37.53,	
Z37.54,	
Z37.59,	
Z37.6,	
Z37.60,	
Z37.61,	
Z37.62,	
Z37.63,	
Z37.64,	
Z37.69,	
Z37.7,	
Z37.9,	
Z38,	
Z38.0,	
Z38.00,	
Z38.1,	
Z38.2,	
Z38.3,	
Z38.30,	
Z38.31,	
Z38.4,	

© 2022 Gordon SH et al. *JAMA Network Open*.

Z38.5,<br>Z38.6,<br>Z38.61,<br>Z38.62,<br>Z38.63,<br>Z38.64,<br>Z38.65,<br>Z38.66,<br>Z38.68,<br>Z38.69,<br>Z38.7,<br>Z38.8	

Table A2.1. Diagnosis and procedure codes used to identify newborn deliveries.

Outpatient visits are defined as all visits with place of service or revenue codes indicating an outpatient setting claim type code, excluding all laboratory claims (place of service code = 81) and any which occurred as part of an emergency department visit or inpatient hospitalization (defined below).

Primary care visits are defined as outpatient visits that included any CPT codes for a preventive office visit (99381-99397), as well as those with ICD-10-CM diagnostic codes for a general adult medical examination (Z00*), other examinations including well woman exams (Z01*), or postpartum care (Z39*) (or corresponding ICD-9-CM codes).

Emergency department Visits are defined using an internal ED flag unique to the CO APCD, as well as claims with the relevant emergency department place of service code (23). We required these claims to have the appropriate revenue codes (0981 or 045X).

Inpatient hospitalizations are defined as all visits which included an admission and discharge date (and where the total length of stay was greater than 24 hours), with appropriate hospitalization point of service or revenue codes listed in Table A2.2

Place of Service Codes	Revenue Codes
21: Inpatient hospital	01XX
51: Inpatient Psychiatric Facility	020X
56: Psychiatric Residential Treatment Center	021X
61: Comprehensive Inpatient Rehabilitation Facility	

Table A2.2. Place of service and revenue codes used to identify inpatient hospitalizations.

© 2022 Gordon SH et al. *JAMA Network Open*.

eAppendix 4

Table A4.1 Health Care Use and Spending Among Individuals in Commercial versus Medicaid Insurance 3-12 Months Postpartum, Estimates Using Untrimmed Spending Sample

	Medicaid enrollment	Commercial enrollment	Unadjusted Difference [95% confidence interval]	Adjusted Difference [95% confidence interval]
N, births	43,400	1,520		
**Patients with Any Utilization, *N* (%)**				
Outpatient Visits	32,073 (73.9%)	1,213 (79.8%)	5.91*** [3.84, 7.97]	7.39*** [6.69, 8.10]
Primary Care Visits	5,069 (11.7%)	231 (15.2%)	3.52*** [1.68, 5.35]	2.68*** [2.34, 3.01]
Emergency Department Visit	12,265 (28.3%)	316 (20.8%)	-7.48*** [-9.56, -5.39]	-7.60** [-8.13, -7.08]
Hospitalizations	1,085 (2.5%)	30 (2.0%)	-0.52 [-1.24, 0.02]	-0.27** [-0.32, -0.22]
**Utilization, Number**				
Outpatient Visits	4.71	4.36	-0.35* [-0.65, -0.05]	0.08** [0.02, 0.14]
Primary Care Visits	0.14	0.20	0.06*** [0.03, 0.08]	0.51*** [0.38, 0.65]
Emergency Department Visits	0.55	0.34	-0.20*** [-0.25, -0.16]	-0.25*** [-0.35, -0.14]
Hospitalizations	0.03	0.03	-0.002 [-0.01, 0.01]	0.02 [-0.31, 0.36]
**Total Spending Per Person**				
Total Spending	$6,927.02	$11,736.71	$4,809.67*** [$1,880.56, $7,738.81]	$6,330.60*** [$4,570.03, $8,091.18]
Outpatient Visits	$2,232.53	$4,781.42	$2,548.90*** [$1,127.81, $3,969.98]	$2,944.54*** [$2,287.92, $3,581.16]
Primary Care Visits	$44.19	$63.86	$19.67 [-$4.51, $43.85]	$35.34** [$3.31, $67.38]
ED visits	$1,588.33	$3,023.77	$1,435.44*** [$745.17, $2,125.71]	$2,065.72*** [$1,070.52, $3,060.93]
Hospitalizations	$2,961.95	$3,763.39	$801.44 [-$1,238.45, $2,884.33]	$337.47 [-$3,392.91, $4,067.85]
**Out-of-Pocket Spending Per Person**				
Total Out-of-pocket	$21.76	$1,270.31	$1,248.55*** [$945.81, $1,551.29]	$1,848.86*** [$1,453.63, $2,266.67]
Outpatient Visits	$8.49	$571.08	$562.59*** [$405.16, $720.02]	$568.26*** [$456.29, $679.59]
Primary Care Visits	$0.14	$5.47	$5.32*** [$2.89, $7.76]	$5.60*** [$2.23, $8.98]
ED visits	$6.19	$454.37	$448.18***	$4,819.27***

© 2022 Gordon SH et al. *JAMA Network Open*.

			[$316.69, $579.66]	[$464.05, $9,174.49]
Hospitalizations	$6.84	$227.84	$221.00*<br>[$8.53, $433.46]	$0.31<br>[-$1.03, $1.64]

Table Notes: Spending is measured in 2020 USD, inflated using the Medical Services Component of the CPI. Utilization outcomes are measured as a binary variable for any visit of that type during the 3-12 month period and in counts at the person level. Spending measures (total and out-of-pocket) are reported as unconditional per-person means over the 3-12 month period. Billed spending measures the sum of insurer and consumer payments. The adjusted differences column reports regression-adjusted coefficients and 95% confidence intervals for each variable. Each coefficient is the estimated coefficient on the treatment dummy (where 1 = continuously enrolled in commercial; 0 = continuously enrolled in Medicaid). Regression covariates include all covariates in Table 1 and year fixed effects. Models are estimated using Newton-Rhapson methods (using the "glm" command in Stata) with a logit link for utilization likelihood (Panel A), a negative binomial distribution and a log link for utilization outcomes (Panel B), and a negative binomial model for spending outcomes (Panel C). Approximate spending changes are presented based on these regression results in Column 5.

* $p < 0.05$, ** $p < 0.01$, *** $p < 0.001$.

© 2022 Gordon SH et al. *JAMA Network Open*.

Table A4.2. Model Comparisons for Health Care Spending Among Individuals in Commercial versus Medicaid Insurance 3-12 Months Postpartum

	Negative Binomial, Untrimmed Sample	Negative Binomial, Trimmed Sample	Gaussian, Trimmed Sample
**Total Spending Per Person**			
Total Spending	\$6,330.60*** [\$4,570.03, \$8,091.18]	\$1,222.50*** [\$544.38, \$1,900.61]	\$1,109.51*** [\$508.91, \$1,710.11]
Outpatient Visits	\$2,944.54*** [\$2,287.92, \$3,581.16]	\$609.27*** [\$302.17, \$916.37]	\$359.73* [\$20.79, \$698.67]
Primary Care Visits	\$35.34** [\$3.31, \$67.38]	\$31.85** [\$1.70, \$61.99]	\$29.09* [\$6.75, \$51.44]
ED visits	\$2,065.72*** [\$1,070.52, \$3,060.93]	\$1,153.86*** [\$456.73, \$1,850.99]	\$1,086.09*** [\$805.27, \$1,366.92]
Hospitalizations	\$337.47 [-\$3,392.91, \$4,067.85]	-\$352.50 [-\$5,373.89, \$4,668.88]	-\$309.17 [-\$637.72, \$19.38]
**Out-of-Pocket Spending Per Person**			
Total Out-of-pocket	\$1,848.86*** [\$1,453.63, \$2,266.67]	\$1,078.03*** [\$848.94, \$1,307.11]	\$795.98*** [\$753.67, \$838.28]
Outpatient Visits	\$568.26*** [\$456.29, \$679.59]	\$393.37*** [\$315.19, \$471.54]	\$361.89*** [\$340.76, \$383.02]
Primary Care Visits	\$5.60*** [\$2.23, \$8.98]	\$5.89*** [\$2.32, \$9.46]	\$5.32*** [\$4.82, \$5.82]
ED visits	\$4,819.27*** [\$464.05, \$9,174.49]	\$4,553.60*** [\$394.75, \$8,712.45]	\$387.28*** [\$359.16, \$415.40]
Hospitalizations	\$0.31 [-\$1.03, \$1.64]	\$0.01 [-\$1.03, \$1.05]	\$34.44*** [\$14.58, \$54.29]

Table Notes: Spending measures (total and out-of-pocket) are reported as unconditional per-person means over the 3-12 month period. Spending is measured in 2020 USD, inflated using the Medical Services Component of the CPI. Billed spending measures the sum of insurer and consumer payments. Each column reports regression-adjusted coefficients and 95% confidence intervals for each variable. Each coefficient is the estimated coefficient on the treatment dummy (where 1 = continuously enrolled in commercial; 0 = continuously enrolled in Medicaid). Regression covariates include all covariates in Table 1 and year fixed effects. Columns 1 and 2 estimate using a negative binomial distribution for spending outcomes, while column 3 estimates the same model using a Gaussian distribution. Columns 2 and 3 exclude the top 1% individuals with the highest levels of billed spending.

* $p < 0.05$, ** $p < 0.01$, *** $p < 0.001$.

© 2022 Gordon SH et al. *JAMA Network Open*.

eAppendix 5. Rates of Missing Covariate Data; Linked Colorado All Payer Claims Database, Birth Records, and Income, 2016-2019

	Medicaid 61-365 days postpartum N missing (%)	Commercial 61-365 days postpartum N missing (%)
Maternal Age	45 (0.1%)	0 (0.0%)
Maternal Race	41 (0.1%)	0 (0.0%)
Maternal Hispanic Ethnicity	41 (0.1%)	0 (0.0%)
Born Outside US	41 (0.1%)	0 (0.0%)
Married	41 (0.1%)	0 (0.0%)
Education		
High School	41 (0.1%)	0 (0.0%)
College	41 (0.1%)	0 (0.0%)
Pre-Pregnancy Chronic Conditions	41 (0.1%)	0 (0.0%)
Number of Prenatal Visits	1,640 (3.8%)	28 (1.9%)
First Trimester Prenatal Care Initiation	41 (0.1%)	0 (0.0%)
Delivery Complications	41 (0.1%)	0 (0.0%)
Cesarean-Section Births	41 (0.1%)	0 (0.0%)
Income at 60 Days Postpartum, % FPL	430 (1.0%)	22 (1.5%)
N, births	42,989	1,482

© 2022 Gordon SH et al. *JAMA Network Open*.
